# Mobile Health Technology Ownership and Use Among Cancer Survivors in a Health System

**DOI:** 10.1002/cnr2.70536

**Published:** 2026-04-02

**Authors:** Shirlene D. Wang, Jing Song, Julia Pincever, Julia Frey, Payton Solk, Kristina Hasanaj, Melanie Wolter, Lauren E. Wang, Fiona Webb, Siobhan M. Phillips

**Affiliations:** ^1^ Department of Preventive Medicine Northwestern University Feinberg School of Medicine Chicago Illinois USA

**Keywords:** cancer survivors, data sharing, digital health adoption, physical activity

## Abstract

**Background:**

Physical activity (PA) is associated with improved health outcomes among cancer survivors (CS), yet PA participation and access to PA programs in cancer care are low. Mobile health (mHealth) technologies such as wearable activity trackers and smartphone lifestyle applications are promising strategies to promote PA among CS. However, CS's adoption patterns and willingness to share resulting mHealth data with healthcare providers are underexplored.

**Aims:**

This study examined mHealth technology ownership and usage as well as willingness to share wearable data with healthcare providers among CS and identified demographic and health‐related correlates.

**Methods:**

Self‐reported data were collected from post treatment CS (*n* = 518; *M*_age_ = 56.5 (SD = 14.5); 54.6% female) from a large healthcare system. Univariate logistic regression models examined associations between demographic (age, sex, race/ethnicity, education, income, marital status, employment status, health status, BMI) and disease (time since diagnosis, treatment received, disease stage) characteristics and meeting PA guidelines (i.e., 150 min/week of moderate to vigorous PA) and activity tracker ownership, lifestyle app usage, and willingness to share wearable data with healthcare providers.

**Results:**

Nearly all CS (97.5%) owned a smartphone. Over half (52.9%) owned an activity tracker, and one‐third (32.4%) used a lifestyle app. Most (64.3%) were willing to share wearable data with healthcare providers. Participants with a college degree or higher income, those who met PA guidelines, and those who were obese were more likely to own a wearable activity tracker. Along with those factors, younger age (< 65) and full‐time employment were also associated with a higher likelihood of using a lifestyle app (*p* < 0.05). Being employed full‐time was significantly associated with willingness to share data with a healthcare provider. No other relationships were significant.

**Conclusions:**

Many CS use or are open to using mHealth technologies. However, differences in adoption by demographic characteristics and unclear demographic and disease correlates of willingness to share data highlight the need for targeted, inclusive, and evidence‐based strategies to integrate these tools into survivorship care. Understanding who adopts mHealth technologies is essential to optimizing their potential to improve long‐term cancer outcomes.

## 1 Introduction

There are an estimated 18.6 million cancer survivors in the United States [1]. This number is expected to increase due to advancements in early detection, treatment, and supportive care. While these advancements have led to increased survival rates, many cancer survivors continue to face long‐term physical, emotional, and psychological challenges [1, 2]. Cancer and its treatments are often accompanied by negative side effects, such as fatigue, pain, and cognitive impairments, which increase the risk for chronic conditions and contribute to a compromised quality of life for survivors [3, 4].

Promoting healthy lifestyle behaviors, particularly regular physical activity (PA), is a crucial strategy for addressing these challenges and improving overall survivorship outcomes. Recently, mobile health (mHealth) technologies, which include smartphone or mobile health applications and wearable devices, have emerged as promising tools to support behavior change in cancer survivors [5]. These technologies can facilitate increased PA by providing real‐time feedback, goal‐setting functions, and progress tracking. Research indicates that mHealth interventions can significantly enhance PA levels, strengthen self‐awareness, and motivate sustained behavior change among survivors [6, 7]. For example, users of health applications and wearables often report higher engagement in moderate‐to‐vigorous physical activity (MVPA), greater frequency of strength training, and improved self‐perceived health status [8].

Importantly, mHealth technologies are becoming increasingly ubiquitous, with growing adoption across various age groups and demographics in the United States. Previous research has indicated that while only 31.8% of cancer survivors reported owning a wearable device, there was a general willingness to share health‐related data, such as blood pressure, heart rate, and PA metrics, with their healthcare providers [6]. This openness to data sharing presents a significant opportunity to integrate mHealth monitoring into clinical care, potentially enabling more personalized survivorship care.

However, the implementation of mHealth technology in cancer survivorship care should address issues of accessibility, equity, and individual preferences. While mHealth has the potential to bridge gaps in healthcare, there may still be hesitancy to adopt this practice. Recent systematic reviews found variability in comfort levels and adoption of health technology among cancer survivors across studies [9, 10]. These differences underscore the need for a more nuanced understanding of who uses mHealth technology and what factors might influence their willingness to share data with researchers and providers. The purpose of this study is to identify more nuanced factors such as demographic characteristics associated with healthy lifestyle app usage, wearable device ownership, and willingness to share activity tracker data with healthcare providers. This study will add to existing literature and inform the development of tailored, accessible, and equitable mHealth interventions that support PA and long‐term well‐being in cancer survivorship.

## 2 Materials and Methods

Data were collected from a cross‐sectional observational study on the interests and preferences of cancer survivors regarding remotely delivered PA interventions between April and June 2023. Participants were recruited from electronic medical records via the Northwestern Medicine Electronic Data Warehouse (NMEDW). Inclusion criteria for the study included self‐reported: (1) ≥ 18 years old; (2) nonmetastatic cancer diagnosis; (3) within 5 years of cancer diagnosis; (4) access to the internet; (5) ability to read and write in English; (6) ≥ 3 months post completion of primary cancer treatment (surgery, radiation, and/or chemotherapy); (7) not currently pregnant. Recruitment efforts aimed to enroll a sample that reflected the national distribution of cancer types, ages, and genders. All participants completed an online informed consent, and the Institutional Review Board at Northwestern University approved all study measures.

### 2.1 Measures

#### 2.1.1 Demographic and Health History

Participants self‐reported age, sex assigned at birth, race/ethnicity, education level, household income, marital status, employment status, health rating, height and weight (to calculate body mass index [BMI]), cancer treatment history (previous chemotherapy or radiation treatment), cancer diagnosis date and age at diagnosis (to calculate time since diagnosis), and cancer type and stage.

#### 2.1.2 Godin Leisure Time Exercise Questionnaire

Weekly minutes of MVPA engagement were collected using a modified version [11] in which participants recalled the number of times per typical week and the average duration (in minutes) for strenuous, moderate, and mild exercise for leisure time PA.

#### 2.1.3 Technology Usage Questionnaire

Participants self‐reported mobile phone ownership [Do you own a mobile phone? (Yes/No); If yes, is this a smartphone (e.g., iPhone, Android)? (Yes/No)], use of healthy lifestyle apps [Do you currently use any healthy lifestyle mobile apps (Yes/No); If yes, which healthy lifestyle apps do you use?], activity tracker ownership [Do you currently own an activity tracker(s)?; (Yes/No) If yes, which activity tracker(s) do you own?] and willingness to share activity tracker data with their healthcare team [If you were to use an activity tracker, would you be willing to have your data shared with your healthcare team (e.g., doctor, nurses, physician's assistant)? (Yes, No, Maybe)]. For those who currently owned an activity tracker, we assessed frequency of wear [How often do you typically wear this activity tracker?] (5–7 days per week; 3–5 days per week; 1–2 days per week; < 1 day per week).

### 2.2 Data Analyses

Frequencies were calculated for all the measures (Table 1). Separate univariate logistic regressions were conducted to examine the associations between demographic and health‐related factors and fitness tracker ownership (yes vs. no), healthy lifestyle app usage (yes vs. no), and willingness to share wearable data with a healthcare provider (yes vs. maybe or no). A binary outcome variable was constructed for the willingness to share outcome, where respondents who answered “yes” were coded as 1 and those who answered “maybe” or “no” were coded as 0, representing certainty versus any hesitancy to share data. Statistical significance was set at *p* < 0.05. Demographic and disease variables examined included: age (≥ 65 vs. <65), sex assigned at birth (female vs. male), race (white vs. non‐white), ethnicity (Hispanic vs. not), education level (< less than college, college, and graduate degree); household income (≥ $100 K vs. < $100 K), marital status (married vs. not), employment status (full time employed vs. not), health rating (poor/fair, good, and very good/excellent), BMI (normal/underweight [BMI < 25], overweight [BMI = 25–30], or obese [BMI > 30]), meeting MVPA guidelines (< 150 min vs. ≥ 150 min per week; [12]), previous chemotherapy, previous radiation, years since diagnosis (> 3 vs. ≤ 3), and cancer stage (Stages 1 or 2 vs. Stages 3 or 4). All analyses were performed using STATA (version 17).

## 3 Results

A total of *N* = 759 participants consented, and *N* = 736 completed a portion of the questionnaire. A total of 518 cancer survivors had complete data for all variables included in these analyses. Sample characteristics are shown in Table 1. Most participants were younger than 65 years (69.50%) (Mean age = 56.52 ± 14.51), female assigned at birth (54.63%), and identified as White (74.13%). Nearly half (49.03%) met the current PA guidelines of at least 150 min per week of MVPA. Most reported undergoing previous chemotherapy (40.35%) or radiation treatment (41.89%). Most participants were diagnosed with early‐stage cancer (75.87%), and the types of cancer are presented in Table 1.

**TABLE 1:** Descriptive statistics, *N* = 518.

	*N* (%)
Younger than 65	360 (69.50%)
Female assigned at birth	283 (54.63%)
White	384 (74.13%)
Hispanic	69 (13.32%)
Education	
Less than college	105 (20.27%)
College	200 (38.61%)
Graduate degree	213 (41.12%)
Income < $100 K	276 (53.28%)
Married	359 (69.31%)
Employed full time	260 (50.19%)
Health rating	
Poor/fair	78 (15.06%)
Good	211 (40.73%)
Very good/excellent	229 (44.21%)
BMI	
Normal/underweight	160 (31.19%)
Overweight	178 (34.70%)
Obese	175 (34.11%)
Meet MVPA guidelines (150 min/week)	254 (49.03%)
Previous chemotherapy	209 (40.35%)
Previous radiation	217 (41.89%)
Time since diagnosis > 3 years	261 (50.39%)
Primary cancer stage	
Early (Stage 1 or 2)	393 (75.87%)
Late (Stage 3 or 4)	125 (24.13%)
Primary cancer type	
Bladder cancer	20 (3.86)
Breast cancer	110 (21.24%)
Colon cancer	30 (5.79%)
Endometrial cancer	33 (6.37%)
Hodgkins's lymphoma	12 (2.32%)
Leukemia/blood cancer	13 (2.51%)
Lung cancer	30 (5.79%)
Melanoma	16 (3.09%)
Non‐Hodgkin lymphoma	21 (4.05%)
Prostate cancer	96 (18.53%)
Renal cancer	22 (4.25%)
Thyroid cancer	24 (4.63%)
Brain cancer	13 (2.51%)
Other	78 (15.06%)
Own a mobile phone	513 (99.05%)
Own a smartphone	505 (97.49%)
Use any healthy lifestyle mobile applications	168 (32.43%)
MyFitnessPal	43 (25.60%)
Nike Training Club	2 (1.19%)
Nike+	5 (2.98%)
Sweat	1 (0.60%)
RunKeeper	5 (2.98%)
Strava	17 (10.12%)
LoseIt!	9 (5.36%)
Weight Watchers (WW)	7 (4.17%)
Noom	11 (6.55%)
I Am	2 (1.19%)
Headspace	7 (4.17%)
Peloton	24 (14.29%)
Tonal	2 (1.19%)
Other	85 (50.60%)
Own an activity tracker	274 (52.90%)
Classic pedometer (step counter)	21 (7.29%)
FitBit	84 (29.17%)
Apple Watch	135 (46.88%)
Garmin watch	24 (8.33%)
WOOP	1 (0.35%)
Oura Ring	4 (1.39%)
Other	48 (16.67%)
Willing to share activity tracker data with healthcare team	
Yes	333 (64.29%)
Maybe	148 (28.57%)
No	37 (7.14%)

Nearly all participants owned a mobile phone (99.05%) and a smartphone (97.49%). However, only 32.43% reported using any healthy lifestyle mobile applications. Among those who reported using healthy lifestyle applications, the most commonly used apps included MyFitnessPal (25.60%), Peloton (14.29%), and Strava (10.12%), with the majority of participants (50.60%) reporting use of other apps not explicitly listed. In contrast, 52.90% of participants reported owning an activity tracker. Among specific devices, the Apple Watch was most commonly reported (46.88%), followed by Fitbit (29.17%). Most participants who owned an activity tracker wore it 5–7 days per week (74.45%). Overall willingness to share data from the activity tracker with healthcare teams was high, with 92.86% responding that they would (64.29%) or might (28.57%). Results of the separate univariate logistic regressions to examine the associations between demographic and health‐related factors and activity tracker ownership, healthy lifestyle app usage, and willingness to share wearable data with healthcare providers are presented in Table 2.

**TABLE 2:** Odds ratios (OR) and 95% confidence intervals (CI) from separate univariate logistic regressions.

	OR (95% CI)
OUTCOME: TRACKER OWNERSHIP (Y/N)	
Younger than 65	1.27 (0.87, 1.84)
Female assigned at birth	1.30 (0.92, 1.83)
White	1.22 (0.82, 1.81)
Hispanic	1.18 (0.71, 1.97)
**College degree (ref. less than college)**	**1.78 (1.10, 2.86)**
Graduate degree (ref. less than college)	1.29 (0.81, 2.07)
**Income > $100 K**	**1.55 (1.09, 2.19)**
**Married**	**2.01 (1.38, 2.94)**
Employed full time	1.18 (0.84, 1.67)
Health rating good (ref. poor/fair)	1.60 (0.95, 2.70)
Health rating excellent (ref. poor/fair)	1.19 (0.72, 2.00)
BMI overweight (ref normal/underweight)	1.33 (0.87, 2.05)
**BMI obese (ref normal/underweight)**	**1.92 (1.24, 2.97)**
**Meet 150 min/week MVPA (Godin)**	**1.53 (1.08, 2.16)**
Previous chemotherapy	0.95 (0.70, 1.35)
Previous radiation	0.94 (0.67, 1.34)
> 3 years since diagnosis	0.94 (0.66, 1.32)
Early‐stage cancer	0.78 (0.52, 1.17)
OUTCOME: HEALTHY LIFESTYLE APP USAGE (Y/N)	
**Younger than 65**	**2.65 (1.69, 4.16)**
Female assigned at birth	1.12 (0.77, 1.62)
White	1.07 (0.70, 1.63)
Hispanic	0.90 (0.52, 1.56)
**College degree (ref. less than college)**	**1.78 (1.04, 3.05)**
**Graduate degree (ref. less than college)**	**1.83 (1.07, 3.13)**
**Income > $100 K**	**1.45 (1.00, 2.11)**
Married	1.26 (0.84, 1.90)
**Employed full time**	**1.51 (1.04, 2.20)**
Health rating good (ref. poor/air)	0.98 (0.57, 1.69)
Health rating excellent (ref. poor/fair)	0.81 (0.47, 1.41)
**BMI overweight (ref normal/underweight)**	**2.16 (1.33, 3.49)**
**BMI obese (ref normal/underweight)**	**2.11 (1.30, 3.43)**
**Meet 150 min/week MVPA**	**1.51 (1.04, 2.18)**
Previous chemotherapy	1.05 (0.72, 1.52)
Previous radiation	1.06 (0.73, 1.54)
> 3 years since diagnosis	0.88 (0.61, 1.27)
Early‐stage cancer	1.25 (0.81, 1.94)
OUTCOME: WILLING TO SHARE WEARABLE DATA WITH HEALTHCARE PROVIDER (YES = 1; NO OR MAYBE = 0)	
Younger than 65	1.11 (0.75, 1.63)
Female assigned at birth	0.79 (0.55, 1.14)
White	0.73 (0.48, 1.12)
Hispanic	0.97 (0.57, 1.65)
College degree (ref. less than college)	0.75 (0.45, 1.24)
Graduate degree (ref. less than college)	0.74 (0.45,1.23)
Income >$100 K	1.25 (0.87, 1.79)
Married	1.09 (0.74, 1.61)
**Employed full time**	**1.44 (1.01, 2.07)**
Health rating good (ref. poor/fair)	0.64 (0.37, 1.13)
Health rating excellent (ref. poor/fair)	0.69 (0.39, 1.21)
BMI overweight (ref normal/underweight)	1.53 (0.98, 2.39)
BMI obese (ref normal/underweight)	1.24 (0.80, 1.94)
Meet 150 min/week MVPA	1.13 (0.79, 1.62)
Previous chemotherapy	1.31 (0.90, 1.90)
Previous radiation	0.89 (0.62, 1.27)
> 3 years since diagnosis	0.78 (0.55, 1.14)
Early‐stage cancer	0.88 (0.57, 1.35)

*Note:* Bolded values are statistically significant.

### 3.1 Activity Tracker Ownership

Univariate analyses revealed that owning an activity tracker was significantly associated with having a college degree, higher income, being married, being in the obesity BMI category, and meeting MVPA guidelines. Specifically, participants with a college degree had higher odds of owning a tracker compared to those with less than a college education (OR = 1.78, 95% CI [1.10, 2.86]). Those with annual household incomes > $100 000 had higher odds compared to those earning less (OR = 1.55, 95% CI [1.09, 2.19]). Being married was associated with more than double the odds of owning a tracker (OR = 2.01, 95% CI [1.38, 2.94]). Obesity was also significantly associated with increased odds of tracker ownership (OR = 1.92, 95% CI [1.24, 2.97]), as was meeting MVPA guidelines (OR = 1.53, 95% CI [1.08, 2.16]).

### 3.2 Healthy Lifestyle App Use

Younger age, higher education, full‐time employment, higher income, overweight/obese BMI, and meeting MVPA guidelines were significantly associated with the use of healthy lifestyle apps. Participants younger than 65 had significantly greater odds of app use (OR = 2.65, 95% CI [1.69, 4.16]). College and graduate degree holders had increased odds compared to those with less than a college education (OR = 1.78, 95% CI [1.04, 3.05] and OR = 1.83, 95% CI [1.07, 3.13], respectively). Full‐time employment was associated with higher odds of app use (OR = 1.51, 95% CI [1.04, 2.20]), as was having an income over $100 000 (OR = 1.45, 95% CI [1.00, 2.11]). Participants who were overweight or obese had more than twice the odds of using healthy lifestyle apps compared to those who were normal weight or underweight (OR = 2.16, 95% CI [1.33, 3.49]; OR = 2.11, 95% CI [1.30, 3.43], respectively). Those meeting MVPA guidelines were also more likely to use healthy lifestyle apps (OR = 1.51, 95% CI [1.04, 2.18]).

### 3.3 Willingness to Share Wearable Data With Healthcare Providers

Only full‐time employment was significantly associated with greater willingness to share wearable data with healthcare providers (OR = 1.44, 95% CI [1.01, 2.07]). No other variables reached statistical significance, although several demonstrated trends that may warrant further investigation. For example, individuals classified as overweight (compared to those with normal or underweight) had marginally higher odds of being willing to share wearable data (OR = 1.53, 95% CI [0.98, 2.39]). Similarly, those with a history of chemotherapy also showed slightly elevated odds (OR = 1.31, 95% CI [0.90, 1.90]).

## 4 Discussion

This study examined the prevalence and correlates of PA mHealth technologies among a large sample of cancer survivors, as well as their willingness to share data from an activity tracker with healthcare providers. These findings contribute to a growing body of literature, suggesting that, while mHealth technology holds substantial promise for supporting PA and survivorship care, there are still differences in adoption and engagement with these tools.

Compared to previous studies, we found higher levels of wearable device ownership in our sample (56% [*N* = 518] vs. 29% [Zhou, *N* = 957], and 41% [Gitonga, *N* = 626]). This is likely due to the increased prevalence and affordability of types of wearables in our recent data collection (2024), compared to 2020–2021 Health Information National Trends Survey (HINTS) data analyzed in those studies [8, 13]. Additionally, our sample was recruited from a healthcare system. The ownership of wearable devices is likely to continue increasing over time, providing an opportunity for integration in care.

Our findings align with previous research indicating that education, income, and PA engagement are key correlates of mHealth technology use [8, 14]. Participants with higher education levels, specifically those with a college degree, were more likely to own an activity tracker and use healthy lifestyle apps. Similarly, survivors with incomes exceeding $100 000 were significantly more likely to report owning a wearable device and had higher odds of using healthy lifestyle apps. This reflects broader socioeconomic trends in mHealth adoption, in which individuals with greater financial and educational resources may have more exposure to, greater comfort with, or easier access to these technologies [15]. Previous studies have also found that younger age, higher education, full‐time employment, higher income, better self‐rated health, and higher health‐related self‐efficacy predict greater use of connected health technologies among cancer survivors [7, 8, 13]. Increasing access across diverse socioeconomic contexts via tracker loan programs, smartphone applications that leverage existing device ownership or include self‐report, smartphone‐integrated accelerometers, and telehealth‐delivered PA interventions could improve technology uptake in those who are socioeconomically disadvantaged. Importantly, meeting current PA guidelines (≥ 150 min/week MVPA) was associated with both activity tracker ownership and healthy lifestyle app use. There is likely a bidirectional reinforcement effect in which engaging with a tracker or lifestyle app is helping cancer survivors meet MVPA guidelines. This also suggests that mHealth technology may be attractive to individuals who are already physically active or motivated to engage in health‐promoting behaviors. Conversely, this raises concerns that mHealth technology may fail to engage survivors who are sedentary or less engaged in self‐management practices, who may benefit the most from PA interventions. To engage these survivors, researchers must consider user needs and motivation. mHealth technology often prioritizes metrics such as step counts, exercise minutes, and activity goals that may feel unattainable for individuals dealing with cancer‐related fatigue, physical limitations, or other psychological barriers to movement. Understanding what metrics are important to these individuals and educating them on how to tailor device metrics to their needs may help increase uptake in these populations. Through techniques such as adaptive goal setting and psychoeducation about PA, fear may be dismantled as a potential barrier to participation. The association between mHealth technology ownership/use and PA levels may also reflect underlying socioeconomic factors discussed above, where lower income correlates with both reduced ownership/use and fewer resources that facilitate PA. Interestingly, BMI also emerged as a significant correlate of mHealth technology use. Individuals who were overweight or obese were more likely to use healthy lifestyle apps and, in the case of those with obesity, more likely to own an activity tracker. This may reflect greater motivation among these individuals to manage weight or monitor health behaviors, given the association between obesity and cancer prognosis for many cancers [16]. It may also indicate exposure to targeted health messaging promoting these technologies, as cancer survivors with overweight and obesity are more likely to receive recommendations about diet and weight and referrals to weight management programs, where they may be exposed to these mHealth technologies [17].

While many participants reported a willingness to share wearable data with their healthcare team, the correlates of this willingness were less clear. Aside from full‐time employment status, no sociodemographic or clinical variables were found to be significantly associated with the outcome. There appears to be openness among survivors to integrating digital monitoring into clinical care; however, we caution that the lack of strong correlates should not necessarily be interpreted as widespread acceptability. Rather, it may reflect factors we did not assess that may influence willingness to share data. Further research is needed to explore additional contextual, psychological, and privacy‐related factors that may influence these decisions. Nonetheless, the observed willingness to share wearable data presents a tangible opportunity for healthcare systems to integrate patient‐generated health data into survivorship care. Several practical strategies could facilitate this integration. First, healthcare systems could establish secure data‐sharing protocols that allow survivors to upload or sync activity tracker data directly into the electronic health record (EHR), either through vendor partnerships with major wearable manufacturers (e.g., Apple Health, Fitbit, Garmin) or through universal health data platforms that aggregate information across devices [18]. This would enable clinicians to review longitudinal PA patterns during routine follow‐up visits, identify trends (e.g., sustained inactivity following treatment), and initiate conversations about barriers to movement or the need for referrals to rehabilitation services. Second, wearable‐derived data could be used to personalize survivorship care plans and tailor PA prescriptions based on individual baseline activity levels, preferences, and functional capacity [19]. For instance, a survivor who demonstrates consistent engagement with moderate‐intensity activity but struggles to meet total weekly minute targets might benefit from structured goal‐setting and incremental challenges, whereas a survivor with minimal activity may require more intensive support such as behavioral counseling. Integration of wearable data could also support remote monitoring programs, where care teams proactively reach out to survivors whose activity levels have declined significantly or who have not met personalized activity targets over a defined period [20]. Such approaches could be particularly valuable for survivors in rural or underserved areas who face barriers to in‐person follow‐up care. Third, healthcare systems could develop dashboards or patient portals that visualize wearable data alongside other clinical metrics (e.g., weight, patient‐reported outcomes, treatment milestones), empowering both survivors and clinicians to collaboratively monitor progress toward health goals [18]. These dashboards could incorporate evidence‐based PA recommendations tailored to cancer type, treatment history, and comorbid conditions, providing context for interpreting activity data. Additionally, automated alerts or nudges could be triggered when activity data suggests potential health concerns, such as prolonged sedentary behavior or abrupt decreases in activity that may signal complications, symptom burden, or psychosocial distress [21]. Finally, clinicians can use wearable data to facilitate more meaningful conversations during clinical encounters [22]. Rather than relying solely on patient recall or subjective assessments of PA, objective data from wearables could serve as a starting point for discussing barriers to movement, setting realistic goals, celebrating progress, and identifying opportunities for intervention. This data‐informed approach may enhance patient engagement and self‐efficacy while also enabling clinicians to provide more personalized, evidence‐based guidance. However, successful integration of wearable data into clinical workflows would require addressing potential issues related to data exchange, clinician training to use the resulting systems, potential algorithmic bias, and the protection of patient privacy and autonomy in data sharing [23].

A strength of our study is the disaggregation of the types of apps and wearables used by survivors. The relatively low adoption rates of structured fitness apps like Nike Training Club or Sweat may suggest that many survivors may not be aware of or may not identify with the branding and functionality of mainstream fitness platforms. Moreover, there is often a cost barrier due to subscription fees associated with these applications. The majority of survivors who reported using an app indicated that they used lifestyle apps not explicitly listed in the survey. Apple Watch and Fitbit were the most popular types of wearable activity trackers. Efforts are underway to integrate data from these widely adopted commercial devices into the EHR, aiming to guide better, more personalized care for survivors [24]. Previous research has shown that wearable activity trackers are acceptable and useful for cancer survivors, with aesthetics being a key factor in determining preference and likelihood of use [25].

The current findings have several practical implications. First, clinicians and survivorship care teams may consider proactively recommending mHealth technology to patients who express interest or already demonstrate some engagement in PA. However, efforts should also focus on reaching underserved populations who may face barriers to access, such as cost, digital literacy, or health literacy so as not to exacerbate existing inequalities. To ensure health inequities are not exacerbated, implementation should be intentionally designed to promote equity. This includes developing low‐cost or free alternatives to commercial wearables, such as smartphone‐based activity tracking applications that include self‐report and leverage devices (i.e., accelerometers embedded in smartphones) that survivors already own, thereby reducing financial barriers. Additionally, interventions should incorporate culturally tailored messaging and multilingual support to enhance relevance and accessibility for diverse populations. Moreover, community‐based partnerships with organizations serving underserved cancer survivors (e.g., safety‐net health systems, community health centers, advocacy groups) could facilitate outreach and engagement with populations less likely to adopt mHealth technologies through traditional clinical channels. Digital health interventions should also be accompanied by digital literacy training and technical support to ensure that survivors with limited technology experience feel empowered to use these tools effectively. Health systems could offer workshops, one‐on‐one coaching, or peer navigator programs to build confidence and competence in using wearable devices and health apps. Importantly, mHealth interventions should not assume universal access to reliable internet connectivity or the latest technology; offline functionality, simplified user interfaces, and compatibility with older devices may be necessary to ensure equitable reach. Second, while most survivors were willing to share their data, ensuring that this data is clinically actionable, secure, and meaningfully integrated into care remains a critical next step. Further qualitative or mixed‐methods research may be needed to explore motivational, privacy‐related, trust in providers, digital literacy, or contextual factors that influence data‐sharing preferences among cancer survivors. Overall, effectively integrating mHealth technology into survivorship care has the potential to maximize the health and well‐being of survivors while mitigating increases in disparities.

This study has limitations worth noting. The sample was recruited from a large health system and was predominantly White, highly educated, higher income, and relatively connected with technology (97% owned smartphones), which may limit generalizability to more diverse populations since wearable usage has been demonstrated to be more likely among women, White individuals, college graduates, and those with annual household incomes greater than $75, 000. Thus, mHealth adoption and willingness to share data patterns in more diverse or underserved survivorship populations may differ and depend on different or more complex socioeconomic and cultural factors [26]. All data were self‐reported, which may introduce reporting bias. Additionally, we did not assess attitudes toward technology, which may mediate adoption and data‐sharing behaviors. Finally, the operationalization of the willingness to share health data variable was limited to categorical responses (Yes/No/Maybe), and a Likert scale would have helped tease out more granularity about perceptions.

Mobile health tools offer promising avenues to support PA and lifestyle management in cancer survivorship. While many survivors use or are open to using these technologies, disparities in adoption and unclear drivers of data‐sharing willingness point to the need for targeted, inclusive, and evidence‐based strategies to integrate these tools into survivorship care. Understanding who adopts mHealth technologies is essential to optimizing their potential to improve long‐term cancer outcomes.

## Author Contributions

Conceptualization: S.D.W., J.S., and S.M.P. Formal analysis: S.D.W. Investigation: J.P. and S.M.P. Writing – original draft: S.D.W. and S.M.P. Writing – review and editing: J.S., J.P., J.F., P.S., K.H., M.W., L.E.W., and F.W. Supervision: J.S. and S.M.P. Funding acquisition: S.M.P.

## Funding

This work was supported by the Department of Preventive Medicine and the Robert H. Lurie Comprehensive Cancer Center (P30CA060553) at Northwestern University Feinberg School of Medicine. Shirlene Wang and Kristina Hasanaj were supported by T32CA193193 and Payton Solk was supported by 1F31CA287985‐01. The content is solely the responsibility of the authors and does not necessarily represent the official views of the National Institutes of Health. This manuscript is the result of funding in whole or in part by the National Institutes of Health (NIH). It is subject to the NIH Public Access Policy. Through acceptance of this federal funding, NIH has been given a right to make this manuscript publicly available in PubMed Central upon the Official Date of Publication, as defined by NIH.

## Ethics Statement

Approval was granted by the Institutional Review Board at Northwestern University.

## Consent

Informed consent was obtained from all individual participants included in the study.

## Conflicts of Interest

The authors declare no conflicts of interest.

## Data Availability

The data that support the findings of this study are available from the corresponding author upon reasonable request.
